# The Human Labial Superficial Musculoaponeurotic System (SMAS): Anatomy, Imaging, and Biomechanical and Sensory Implications—A Narrative Review

**DOI:** 10.3390/diagnostics16193208

**Published:** 2026-10-02

**Authors:** José Martín-Cruces, Estefanía del Rocío Ocampo Poma, Luis Alberto Chauca Bajaña, Mirian Teulé-Trull, Patricia Cuendias, Yolanda García-Mesa, José A. Vega

**Affiliations:** 1 Master ENDORE, Departamento de Cirugía y Especialidades Médico-Quirúrgicas, Universidad de Santiago de Compostela, 15705 Santiago de Compostela, Spain; jose.martin.cruces@usc.es (J.M.-C.); miriam.teule.trull@usc.es (M.T.-T.); 2 Facultad de Odontología, Universidad Católica de Santiago de Guayaquil, Guayaquil 090101, Guayas, Ecuador; estefania.ocampo@cu.ucsg.edu.ec (E.d.R.O.P.); luis.chaucab@ug.edu.ec (L.A.C.B.); 3 Cátedra Hispanoamericana de Investigación en Implantología Clínica y Prótesis Dental, Facultad de Ciencias de la Salud, Universidad Católica de Santiago de Guayaquil–SOADCO, Guayaquil 090101, Guayas, Ecuador; 4 Grupo de Investigación de Prevención y Salud Oral, Universidad Católica de Santiago de Guayaquil, Guayaquil 090101, Guayas, Ecuador; 5 Facultad de Odontología, Universidad Bolivariana del Ecuador, Durán 092406, Ecuador; 6 Facultad de Odontología, Universidad de Guayaquil, Guayaquil 090101, Guayas, Ecuador; 7 Departamento de Morfología y Biología Celular, Grupo SINPOS, Universidad de Oviedo, Oviedo, Spain; cuendiaspatricia@uniovi.es (P.C.); garciamyolanda@uniovi.es (Y.G.-M.); 8 Instituto de Investigación Sanitaria del Principado de Asturias, 33011 Oviedo, Spain; 9 Facultad de Ciencias de la Salud, Universidad Autónoma de Chile, Santiago, Chile

**Keywords:** superficial musculoaponeurotic system, SMAS, lips, orbicularis oris, connective tissue, facial anatomy, perioral anatomy, lip anatomy, fibromuscular connective tissue, vermilion, aesthetic surgery, reconstructive surgery

## Abstract

The superficial musculoaponeurotic system (SMAS) is a fundamental concept in facial anatomy and surgery, but its organization within the human lips remains incompletely defined. This narrative review critically reassesses the anatomical evidence for a labial SMAS and its regional configuration. Published anatomical, histological, and imaging evidence was critically reviewed. The literature review is complemented by original qualitative histological and imaging observations of the human lips, included exclusively for morphological correlation with the published anatomical evidence and not as an independent quantitative study. The available evidence does not demonstrate a classical, readily separable laminar SMAS within the lips. Instead, it supports a three-dimensional connective tissue network extending from the oral mucosa to the skin and intimately incorporating the orbicularis oris muscle. This organization is associated with neurovascular structures and may have biomechanical and sensory implications, although these functional interpretations are not directly demonstrated by the present material. The labial connective tissue architecture differs from the classical laminar SMAS described in other facial regions and may represent its regional equivalent. Further three-dimensional anatomical studies are required to establish this interpretation conclusively. By defining the normal fibromuscular architecture of the lips and the limits of its visualization, this review provides an anatomical framework for interpreting lip imaging while emphasizing that a labial SMAS is not currently a validated diagnostic or prognostic marker.

## 1. Introduction

The superficial musculoaponeurotic system (SMAS), originally described by Mitz and Peyronie in 1976 [1], is a central concept in facial anatomy and surgery. Subsequent studies have shown that its morphology varies substantially among facial regions [2]. The lips are particularly complex because the orbicularis oris, connective tissue, skin, vermilion, and oral mucosa are closely integrated within a highly mobile and richly innervated structure.

Whether the lips contain a regional equivalent of the SMAS remains unresolved. Published anatomical studies describe connective tissue relationships between the orbicularis oris and the superficial tissues, but the available evidence is limited, methodologically heterogeneous, and does not demonstrate a classical, readily separable SMAS layer. The principal anatomical question is therefore how the connective tissue is organized through the thickness of the lip and whether that organization can reasonably be interpreted as a regional expression of the facial SMAS.

The aim of this narrative review is to critically reassess the published anatomical, histological, and imaging evidence concerning the labial SMAS, distinguishing direct morphological observations from biomechanical or sensory interpretations. Original qualitative histological and imaging observations from our collections are included only for morphological correlation with the published evidence.

## 2. Materials and Methods

### 2.1. Literature Search Strategy

A narrative literature search was performed in PubMed, Scopus, and Google Scholar and updated through 18 September 2026. Search terms included “labial SMAS”, “superficial musculoaponeurotic system”, “lip anatomy”, “labial connective tissue”, “orbicularis oris”, “lip histology”, “lip imaging”, “labial artery”, “facial nerve”, “lip aging”, and related combinations. Studies were selected for their direct relevance to the anatomy, histology, imaging, neurovascular relationships, aging, or clinical interpretation of the human lips and facial SMAS. A narrative approach was considered appropriate because the available literature is limited and methodologically heterogeneous, comprising cadaveric dissections, histological studies, imaging investigations, and conceptual anatomical analyses that are not directly amenable to quantitative synthesis. No formal systematic review or quantitative study-selection procedure was applied, and no retrospective record count is reported because such a count was not prospectively recorded.

### 2.2. Human Tissue Material and Histological Procedures

Human tissue material consisted of paired upper- and lower-lip specimens from six subjects (four women and two men; age range, 46–72 years), providing 12 samples. Full-thickness sagittal sections from the central region of the upper and lower lips were examined. Representative sections stained with Masson’s trichrome and sections processed for CD34 and vimentin immunohistochemistry were evaluated qualitatively. The vimentin-stained sections belonged to the same research collection and were used only for qualitative identification of fibroblastic cell profiles. The fixation, tissue processing, sectioning, staining, immunohistochemical procedures, antibody validation, and controls used for this research collection have been described in detail in our previous studies of human lip innervation [3,4]. The present observations were not designed as an independent quantitative study and were included exclusively for morphological correlation with the literature reviewed here.

### 2.3. Imaging Material

Representative magnetic resonance and ultrasound images of the perioral region were selected from the pre-existing anonymized teaching image archive of the Department of Morphology and Cell Biology, Anatomy Section, University of Oviedo. No imaging examinations were performed specifically for this review. Because the material derives from a teaching archive rather than a prospectively designed imaging study, acquisition protocols were not standardized. The exact number of individuals represented, the original ultrasound probe orientation, and the complete acquisition parameters cannot be recovered from the archive records and are therefore explicitly reported as unavailable. The magnetic resonance examples shown are axial images. Images were selected solely to demonstrate the spatial relationship of the orbicularis oris with adjacent soft tissues and are presented only as qualitative morphological correlation, not as independent evidence for a labial SMAS.

## 3. Current Evidence Supporting the Existence of a Human Labial SMAS

### 3.1. Historical Background

The concept of the SMAS was originally introduced by Mitz and Peyronie in 1976 [1] following their anatomical investigations of the parotid and cheek regions. Their description of a continuous fibromuscular layer interposed between the facial mimetic muscles and the overlying skin rapidly transformed the understanding of facial anatomy and provided the anatomical basis for many contemporary facial surgical procedures. Since then, the SMAS has become one of the central concepts in facial anatomy, although its structural organization has subsequently been shown to vary substantially among different facial regions [2,5].

Despite the extensive literature devoted to the facial SMAS, the lips have received comparatively little attention. Their unique anatomical organization, characterized by the intimate integration of skeletal muscle, connective tissue, dermis, vermilion and oral mucosa, differs from that of regions where the classical concept of the SMAS was originally established. Accordingly, whether the connective tissue organization of the lips should be regarded as a regional specialization of the facial SMAS or as a distinct anatomical entity remains unresolved [6].

### 3.2. The First Anatomical Evidence

The first direct anatomical evidence supporting the existence of a SMAS within the human lips was provided by Pensler, Ward and Parry in 1985 [7]. Their investigation represented a turning point in the anatomical interpretation of the perioral region because the connective tissue of the upper lip was examined within the conceptual framework of the SMAS. Rather than considering the perioral connective tissue as part of the ordinary subcutaneous layer, the authors proposed that it constituted an organized fibromuscular system establishing continuity between the orbicularis oris and the overlying dermis. The study combined meticulous anatomical dissection with histological examination, allowing macroscopic observations to be correlated with the microscopic organization of the tissues. The dissections demonstrated a fibromuscular connective tissue layer situated superficial to the orbicularis oris, while histological analysis confirmed the presence of collagen fibres, elastic fibres and skeletal muscle fibres extending towards the dermis.

A key contribution of Pensler et al. [7] was the recognition that this connective tissue organization could provide an anatomical substrate for the transmission of muscular forces from the orbicularis oris to the superficial tissues.

Although pioneering, the study had several methodological limitations, including a relatively small cadaveric series, the absence of quantitative morphometric analysis, and the inability to reconstruct the three-dimensional architecture of the connective tissue network. Moreover, interpretation of the findings was necessarily influenced by the original concept of the SMAS derived from the lateral face, whose connective tissue architecture differs substantially from that of the lips. Despite these limitations, the work of Pensler et al. [7] introduced the concept that the connective tissue of the lips should be interpreted as an organized fibromuscular system rather than as undifferentiated subcutaneous connective tissue.

### 3.3. Functional Interpretation of the Labial Connective Tissue

Following the study of Pensler et al. [7], Jost, Levet and Wassef [8] published a brief letter commenting on the concept of a superficial musculoaponeurotic system in the upper lip. The publication supported interpreting the connective tissue associated with the orbicularis oris within the SMAS framework, but its format provides limited methodological information.

Because sample characteristics, anatomical procedures, and histological methods were not reported with the detail of a full anatomical study, this letter cannot be treated as an independently assessable demonstration of a continuous three-dimensional labial network.

Its principal contribution was interpretative: it emphasized a possible functional relationship between the fibromuscular connective tissue and the dynamic activity of the orbicularis oris. Proposed roles in force transmission, coordination of superficial tissue deformation, and maintenance of lip contour were not experimentally demonstrated in that publication.

Accordingly, the observations and interpretation offered in the letter should be distinguished from direct biomechanical evidence. The publication is relevant to the historical development of the labial SMAS concept, but it does not have the evidential weight of a fully reported anatomical or experimental investigation.

**Figure 1:**
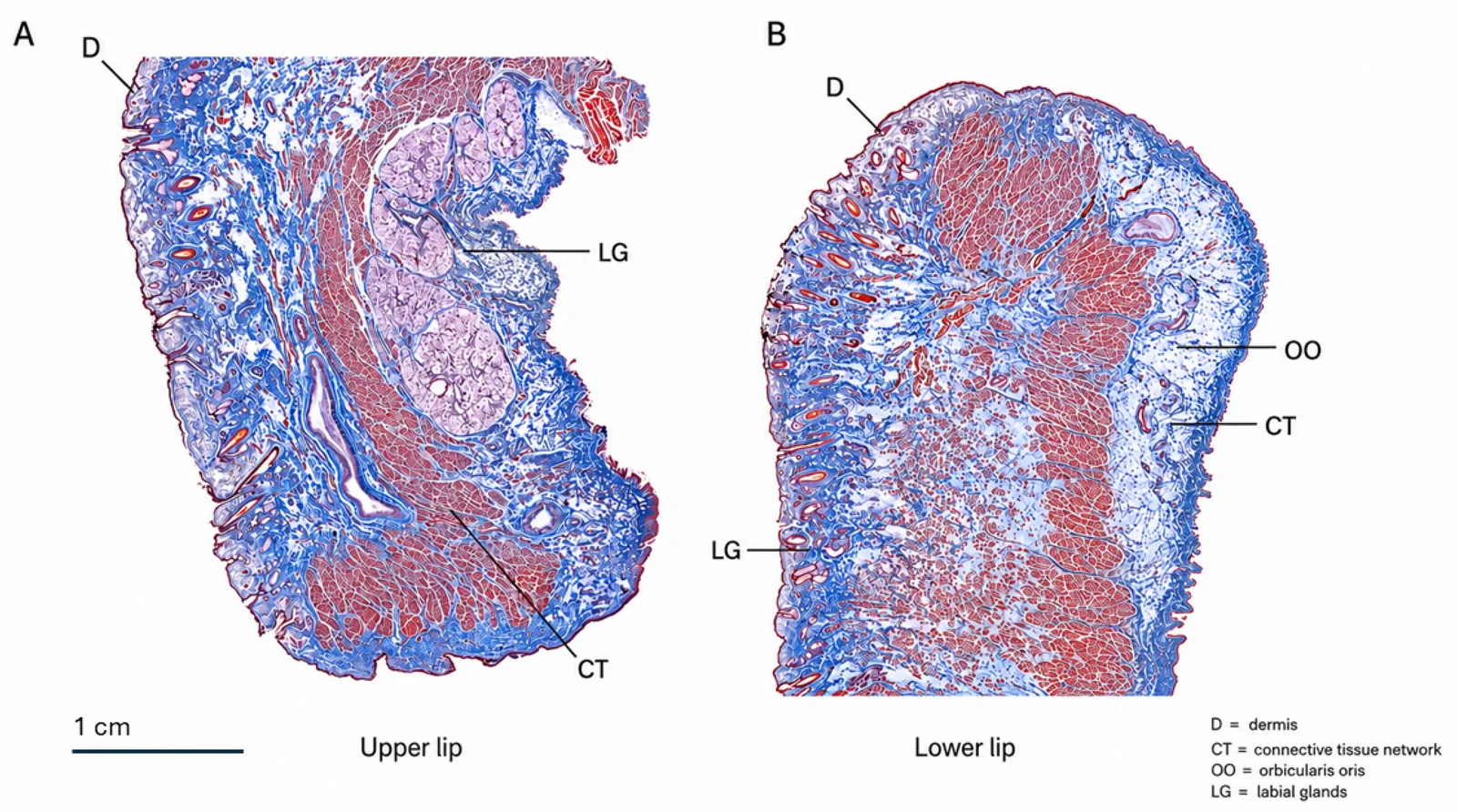
Full-thickness sagittal histological sections from the central regions of the human upper (**A**) and lower (**B**) lips stained with Masson’s trichrome. The orbicularis oris muscle is stained red, whereas the collagen-rich connective tissue is stained blue. A continuous connective tissue network surrounds and penetrates the muscular fascicles and extends towards the superficial tissues of the lip. D, dermis; CT, connective tissue network; OO, orbicularis oris; LG, labial glands. Scale bar, 1 cm. Images are derived from original, previously unpublished human tissue material from the authors’ research collection (SINPOS Research Group—University of Oviedo).

Qualitative observations from human lip specimens belonging to our laboratory research collection provide additional morphological correlation with these published findings. Histological examination shows that the endomysial and perimysial connective tissue of the orbicularis oris is continuous with a network of connective tissue septa extending throughout the thickness of the lips towards the dermis, vermilion, and oral submucosa (Figure 1). These septa contain blood vessels of different calibres and peripheral nerves, including branches contributing to perivascular plexuses, motor innervation of the muscle, and sensory nerve structures within the vermilion and submucosa. The methodological and ethical details concerning this original material are provided in the corresponding Section 2.

The contribution of Jost et al. [8] is therefore retained as an influential historical interpretation, while its publication type and methodological limitations are made explicit.

### 3.4. Regional Specialization of the Facial SMAS

The anatomical studies published by Ghassemi et al. [9] represented a major conceptual advance in the understanding of the SMAS. Unlike previous investigations, which sought to identify the SMAS as a distinct anatomical layer, these authors proposed that its architecture varies substantially according to the anatomical region examined. Accordingly, the SMAS should be regarded as a regionally specialized connective tissue system instead of a morphologically uniform structure throughout the face.

Using anatomical dissection combined with conventional histology, polarized-light microscopy, and confocal microscopy, Ghassemi et al. [9] examined multiple facial regions and demonstrated marked regional differences in connective tissue organization. Based on these observations, they proposed two principal architectural patterns. Type I, characteristic of the lateral face, consisted of fibrous septa surrounding adipose lobules, whereas Type II, observed in the perioral region, was characterized by a dense fibromuscular network containing relatively little adipose tissue and a close interrelationship between collagen fibres, elastic fibres and skeletal muscle.

This classification is particularly relevant for understanding the anatomy of the lips. In the perioral region, the connective tissue does not form an isolated fibrous membrane that can be easily separated from the underlying musculature. Instead, connective tissue fibres interdigitate with the orbicularis oris and extend towards the dermis and vermilion, creating a compact three-dimensional framework. Accordingly, the absence of a clearly defined fascial plane should not be interpreted as evidence against the existence of a labial SMAS but rather as a reflection of the distinctive structural organization of the lips.

Another notable contribution of Ghassemi et al. [9] was the recognition that regional anatomical specialization is closely related to regional function. The connective tissue architecture observed in the lips reflects the exceptional mechanical demands imposed by continuous movement.

Although the study did not focus exclusively on the lips, its conceptual contribution has had considerable influence on subsequent investigations of the perioral region. By emphasizing regional specialization rather than morphological uniformity, Ghassemi et al. established an anatomical framework within which the connective tissue organization of the lips could be interpreted without requiring strict correspondence with the classical SMAS described in the parotid and cheek regions.

### 3.5. Integration of Anatomy, Histology and Imaging

The work of Hînganu et al. [10] represents one of the most comprehensive investigations performed to date on the connective tissue organization of the human lips. Unlike previous studies, these authors adopted a multimodal approach combining gross anatomy, histology, and radiological imaging. This integrated methodology provided the opportunity to correlate anatomical observations obtained from cadaveric specimens with the appearance of the perioral soft tissues in vivo.

Meticulous layer-by-layer dissections demonstrated that the superficial fascia continues towards the oral region, where its morphology gradually changes as it approaches the lips. The labial connective tissue progressively intermingles with the orbicularis oris, becoming incorporated into a dense fibromuscular framework extending throughout both the upper and lower lips. Histological examination confirmed this close structural relationship, showing abundant collagen fibres surrounding muscular fascicles and establishing continuity with the dermis and the connective tissue of the vermilion.

A notable feature of this investigation was the incorporation of magnetic resonance (MR) imaging. This represented an important methodological advance because it linked classical anatomy with contemporary imaging and opened new possibilities for investigating the connective tissue architecture of the lips under physiological conditions.

They also described the presence of an infra-SMAS compartment within the perioral region. Its description emphasizes the complexity of the connective tissue organization surrounding the orbicularis oris and illustrates that the lips cannot be adequately interpreted using simplified layer-based anatomical models. Instead, the connective tissue forms a continuous three-dimensional framework containing muscular, vascular, and connective tissue components arranged according to regional functional requirements.

Perhaps the principal contribution of Hînganu et al. [10] was the demonstration that the question of whether the lips possess a SMAS cannot be answered simply by identifying or failing to identify a discrete fascial layer. Their observations indicate that the superficial fascia undergoes a gradual structural transformation as it approaches the lips, becoming integrated within the specialized connective tissue architecture of the perioral region.

Collectively, these studies demonstrate a substantial progression in anatomical understanding. While each study approached the problem from a different perspective, all consistently recognized the presence of an organized fibromuscular connective tissue framework associated with the orbicularis oris (Table 1).

## 4. Proposed Interpretative Criteria for a Regional Labial SMAS Equivalent

One of the principal difficulties in interpreting the connective tissue organization of the lips lies in the absence of universally accepted anatomical criteria defining a regional labial SMAS equivalent. The criteria discussed below are therefore proposed interpretative criteria and not validated diagnostic criteria demonstrating the existence of a labial SMAS.

The original concept proposed by Mitz and Peyronie described the SMAS as a continuous fibromuscular layer interposed between the facial mimetic muscles and the overlying skin. This organization established structural continuity between muscle and dermis and was considered responsible for transmitting muscular forces to the superficial soft tissues.

The mere presence of connective tissue surrounding or traversing muscle is not sufficient to classify that tissue as SMAS, because endomysial, perimysial, and ordinary subcutaneous connective tissues also integrate muscle with adjacent structures. A regional SMAS equivalent would require a reproducible, organized fibromuscular continuum linking the orbicularis oris with superficial tissues across the lip and showing topographic continuity with the superficial facial fascial system, rather than only the local supportive connective tissue expected around skeletal muscle.

When these proposed principles are applied to the lips, the published observations are more compatible with a dense three-dimensional fibromuscular organization than with an isolated fascial plane. This anatomical organization is directly observable; its classification as a regional SMAS equivalent remains the interpretative hypothesis evaluated in this review.

Accordingly, the proposed interpretative features are: (1) a reproducible and organized fibromuscular connective tissue continuum; (2) structural continuity between connective tissues associated with the orbicularis oris and the superficial tissues; (3) integration of collagen bundles, elastic fibres and connective tissue septa into a three-dimensional network extending across the lip; and (4) demonstrable topographic continuity with the superficial facial fascial system. These features distinguish the hypothesis of a regional SMAS equivalent from the nonspecific presence of ordinary muscle-associated connective tissue, but they do not by themselves validate that classification.

## 5. Structural Organization of the Labial Connective Tissue

The anatomical studies published to date consistently support the presence of an organized fibromuscular connective tissue framework within the lips, while also demonstrating that its organization differs substantially from the classical description of the SMAS in the lateral face [7,8,9,10].

This complex organization reflects the unique anatomical requirements of the perioral region. The orbicularis oris muscle occupies the central core of the lips and lies in close proximity to both the dermis and the oral mucosa. Accordingly, the connective tissue framework does not simply surround the muscle but penetrates and interconnects its fascicles, extending superficially towards the dermis and deeply towards the lamina propria of the oral mucosa. Numerous fibrous septa establish continuity between these different tissue compartments, producing a highly integrated structural organization rather than a succession of independent anatomical layers.

Another important characteristic is the intimate relationship between the connective tissue and the vermilion. Unlike ordinary skin, the vermilion lacks cutaneous appendages and possesses a highly specialized connective tissue stroma rich in vascular papillae and collagen bundles. These connective tissue components contribute not only to the characteristic morphology of the vermilion border but also to its mechanical behaviour during lip movement. Their continuity with the connective tissue surrounding the orbicularis oris further supports the concept that the lips should be regarded as a single fibromuscular organ.

The connective tissue framework also serves as the principal pathway for the neurovascular supply of the lips. Branches of the superior and inferior labial arteries, accompanying veins, lymphatic vessels, and terminal sensory nerves travel within fibrovascular septa before ramifying throughout the superficial and deep tissues. This anatomical arrangement allows the connective tissue to function simultaneously as a mechanical support, an organizational scaffold, and a conduit for the vascular and neural elements essential for normal lip physiology.

**Figure 2:**
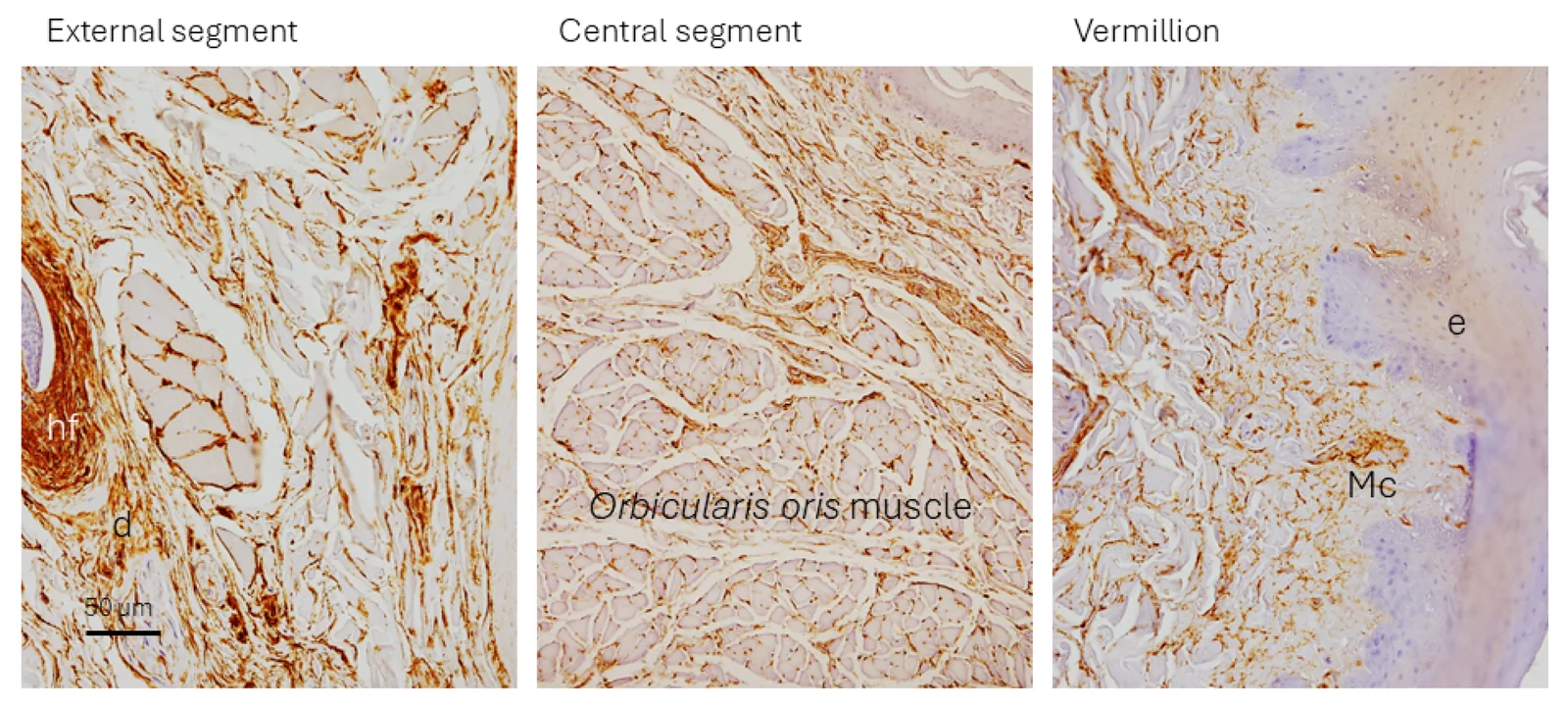
Full-thickness sagittal section of the central region of the human upper lip processed for CD34 immunohistochemistry, demonstrating CD34-positive stromal cells within the connective tissue. CD34 immunoreactivity is visualized in brown. In the central panel, the endomysial and perimysial connective tissue surrounds the orbicularis oris muscle fibres and continues with connective tissue septa of different calibres. This cellular network extends continuously towards the dermis and hair follicles (left) and towards the vermilion (right). In the vermilion, the CD34-positive network is continuous with the CD34-positive connective tissue capsule of Meissner corpuscles. CD34 immunoreactivity alone is not considered sufficient to establish a telocyte phenotype. HF, hair follicle; MC, Meissner corpuscle; e, vermilion epithelium. Scale bar, 50 µm. Images are derived from original, previously unpublished human tissue material from the authors’ research collection (SINPOS Research Group—University of Oviedo).

Original qualitative immunohistochemical observations from human lip specimens belonging to our laboratory research collection provide additional morphological evidence of the cellular organization of the labial connective tissue framework. In addition to its extracellular matrix components, this framework contains an extensive network of connective tissue cells, including vimentin-positive fibroblastic cells and CD34-positive stromal cells [11] (Figure 2). These cellular elements form a continuous network associated with the endomysial and perimysial connective tissue of the orbicularis oris and with connective tissue septa extending towards the dermis and vermilion. At the vermilion, this CD34-positive connective tissue network is closely associated with sensory corpuscles, where CD34 immunoreactivity is also observed in the outer connective tissue capsule. No telocyte phenotype is inferred from the present CD34 immunoreactivity. The methodological and ethical details concerning this original material are provided in the corresponding Section 2.

## 6. Functional Anatomical Significance of the Labial Connective Tissue Framework

The structural organization of the connective tissue within the lips is closely related to the unique functional demands imposed upon the perioral region. Unlike other facial areas, where soft tissue movement is often localized, the lips undergo continuous and highly coordinated deformation during speech, mastication, swallowing, facial expression, and oral competence. These functions require not only coordinated activity of the orbicularis oris and other perioral muscles but also the transmission and distribution of mechanical forces throughout the surrounding soft tissues.

The observed connective tissue relationships provide an anatomical basis from which mechanical integration may be hypothesized [12]. Fibrous connections between the orbicularis oris and adjacent tissues could contribute to the distribution of deformation across the lip, particularly at the vermilion border. However, the present review includes no direct biomechanical measurements; force transmission, tissue adaptation and related functional effects should therefore be regarded as biomechanical implications of the anatomy rather than experimentally demonstrated functions.

The connective tissue is also closely related to the neurovascular anatomy of the lips. The orbicularis oris receives motor innervation principally from the buccal and marginal mandibular branches of the facial nerve. The superior and inferior labial arteries show marked variability in their depth relative to the orbicularis oris, with submucosal and intramuscular courses predominating and subcutaneous courses being less frequent [13,14,15]. Sensory nerves and mechanoreceptors are distributed through the dermis, vermilion, and mucosa [3,4]. These relationships are clinically relevant, but they do not establish that vessels or nerves occupy a constant plane within a putative labial SMAS.

## 7. Biological Significance of the Labial Fibromuscular Connective Tissue System

Connective tissue is biologically active and undergoes continuous extracellular-matrix turnover in response to cellular and mechanical signals. Fibroblasts and myofibroblasts participate in matrix production, remodeling, and mechanotransduction [16,17,18]. In the present material, CD34-positive cells are described conservatively as stromal cells; no specific telocyte phenotype or mechanobiological function is inferred from CD34 immunoreactivity alone.

The perioral region is exposed to an exceptional range of functional demands throughout life. Every movement associated with speech, mastication, swallowing, facial expression or oral competence generates repetitive mechanical forces that must be distributed efficiently throughout the tissues without compromising structural integrity. This biological interpretation is consistent with the histological organization of the lips. The intimate relationship between collagen bundles, elastic fibres, muscular fascicles and fibrovascular septa suggests that connective tissue contributes not only to tissue cohesion but also to the regulation of tissue mechanics. Continuous remodelling of the extracellular matrix, together with the intrinsic mechanical properties of collagen and elastic fibres, allows the lips to maintain their structural stability while preserving the flexibility required for highly coordinated movement.

An additional aspect deserving consideration is the close relationship between connective tissue and the rich sensory innervation of the lips [3,4]. Sensory nerve branches course within the connective tissue framework before terminating within the dermis, vermilion and oral mucosa. Although the available evidence does not permit definitive conclusions regarding functional interactions between the connective tissue framework and sensory receptors, their close anatomical relationship—including the continuity observed between the labial connective tissue network and the connective tissue capsule of sensory corpuscles—raises the possibility that tissue deformation may influence the mechanical environment in which these receptors operate. This interpretation remains a biological hypothesis rather than an established functional mechanism.

Taken together, these observations indicate that the connective tissue of the lips is structurally integrated with muscle, vessels, nerves and superficial tissues. Its possible contributions to mechanical behavior and sensory function remain plausible interpretations that require direct experimental testing.

## 8. Diagnostic, Prognostic and Clinical Relevance

A detailed understanding of labial anatomy is relevant to diagnostic interpretation, reconstructive and aesthetic procedures. The variable relationships among the orbicularis oris, labial arteries and connective tissues should be considered during surgical dissection, reconstruction and minimally invasive procedures [13,19]. The present review does not compare procedural techniques and therefore does not prescribe specific injection depths or surgical approaches.

Rather than assuming a constant laminar SMAS plane within the lips, clinicians should recognize a close three-dimensional relationship between muscle and connective tissues extending from the mucosa to the skin. This interpretation may help explain why tissue behavior during dissection or augmentation differs from that of facial regions in which a more readily separable SMAS layer is present.

For injectable procedures, the marked variability of the superior and inferior labial arteries relative to the orbicularis oris is particularly relevant [13]. Recent high-frequency ultrasound also shows that lip-layer thickness and filler distribution vary among individuals [20]. These findings support anatomically informed caution but do not justify a single universally safe injection plane.

For reconstructive procedures, preservation or accurate restoration of the relationships among the orbicularis oris, vermilion, skin and mucosa is anatomically important for maintaining contour and mobility. The present evidence supports attention to these relationships without implying that a discrete SMAS layer can always be surgically identified within the lip.

### 8.1. Diagnostic Significance

Knowledge of the normal integrated mucosa-to-skin architecture of the lip provides an anatomical reference for interpreting magnetic resonance and high-frequency ultrasound images. These modalities can depict the orbicularis oris, gross soft-tissue compartments, vascular relationships, lip-layer thickness and filler distribution [13,20,21,22], but the selected evidence does not support reliable identification or exclusion of a microscopic labial SMAS as a separate imaging structure. Consequently, the proposed regional SMAS equivalent should not be used as an imaging diagnostic criterion. Its significance to the field of diagnosis lies in providing a more accurate normal anatomical framework for interpreting lip morphology and procedure-related imaging findings. The present review does not evaluate diagnostic sensitivity, specificity or accuracy for any disease.

### 8.2. Prognostic Relevance

Current evidence does not establish the presence, thickness or configuration of a labial SMAS as an independent predictor of functional recovery, complication risk, aesthetic durability or treatment response. Any prognostic relevance is therefore indirect and anatomical: preservation or restoration of the relationships among the orbicularis oris, vermilion, skin, mucosa and vascular structures may be important for postoperative contour and mobility, but outcome-linked prospective studies are required before these anatomical features can be assigned prognostic value [19,23,24,25,26,27,28].

Age-related structural changes in the lips are documented histologically. Penna et al. demonstrated significant thinning of both the cutis and orbicularis oris in older individuals, together with degeneration of collagen and elastic fibres [28]. More recent high-frequency ultrasound data indicate that lip anatomy also varies with age, sex, race and ethnicity, and body mass index [20]. These observations support demographic variability in labial anatomy, but the current evidence is insufficient to define consistent population-specific differences in a putative labial SMAS.

Importantly, the purpose of this review is not to establish recommendations regarding specific surgical techniques or aesthetic procedures. Rather, it is to provide the anatomical framework upon which such procedures should be based. A precise understanding of the connective tissue organization of the lips enables reconstructive surgeons, oral and maxillofacial surgeons, dentists involved in perioral aesthetics and physicians performing aesthetic medicine to interpret tissue behaviour more accurately and to perform anatomically guided interventions with a clearer appreciation of the structural complexity of the perioral region.

## 9. Discussion

The principal objective of this review was to critically evaluate whether the connective tissue organization of the human lips fulfils the anatomical criteria supporting the concept of a labial superficial musculoaponeurotic system. Although relatively few studies have specifically addressed this question, their fundamental morphological observations are remarkably consistent. Collectively, they demonstrate an organized connective tissue architecture intimately associated with the orbicularis oris and continuous with the superficial tissues of the lips.

The principal studies supporting a labial SMAS must be interpreted in light of their methodological limitations. Pensler et al. [7] provided direct cadaveric evidence but studied a limited series and could not reconstruct the three-dimensional architecture. Jost, Levet and Wassef [8] contributed an influential anatomical interpretation, but the publication was brief and did not provide the methodological depth of a full anatomical study. Ghassemi et al. [9] established the importance of regional SMAS variation but did not focus exclusively on the lips. Hînganu et al. [10] combined dissection, histology and imaging, although the sample remained limited. These studies are complementary, but no single investigation provides definitive evidence for a discrete labial SMAS.

The anatomical evidence is more consistent with a three-dimensional connective tissue network extending from the oral mucosa to the skin and intimately incorporating the orbicularis oris than with a classical, readily separable fascial lamina. We therefore do not define the labial SMAS simply as any organized connective tissue network. Instead, we describe the anatomy independently and propose that this distinctive mucosa-to-skin organization may represent the regional equivalent of the SMAS in the lips.

**Figure 3:**
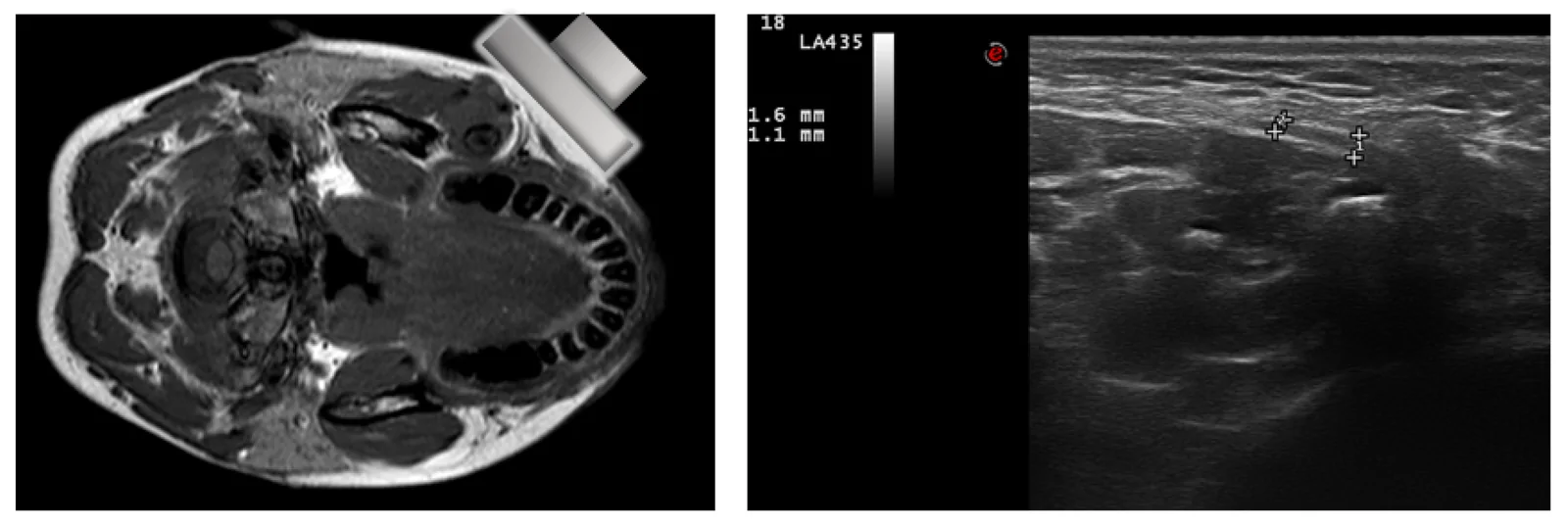
Representative axial magnetic resonance image (left) and ultrasound image (right) of the upper lip. The position indicated on the magnetic resonance image corresponds to the approximate placement of the ultrasound transducer used to obtain the sonographic plane shown on the right. The orbicularis oris occupies the central muscular core and is closely integrated with the adjacent superficial and mucosal soft tissues. A separate SMAS-like layer is not resolved on these selected illustrative images. Images are original, previously unpublished, anonymized images selected from the teaching image archive of the Department of Morphology and Cell Biology, Anatomy Section, University of Oviedo, and were not obtained specifically for the present study. The original ultrasound probe orientation and complete acquisition parameters are unavailable.

**Figure 4:**
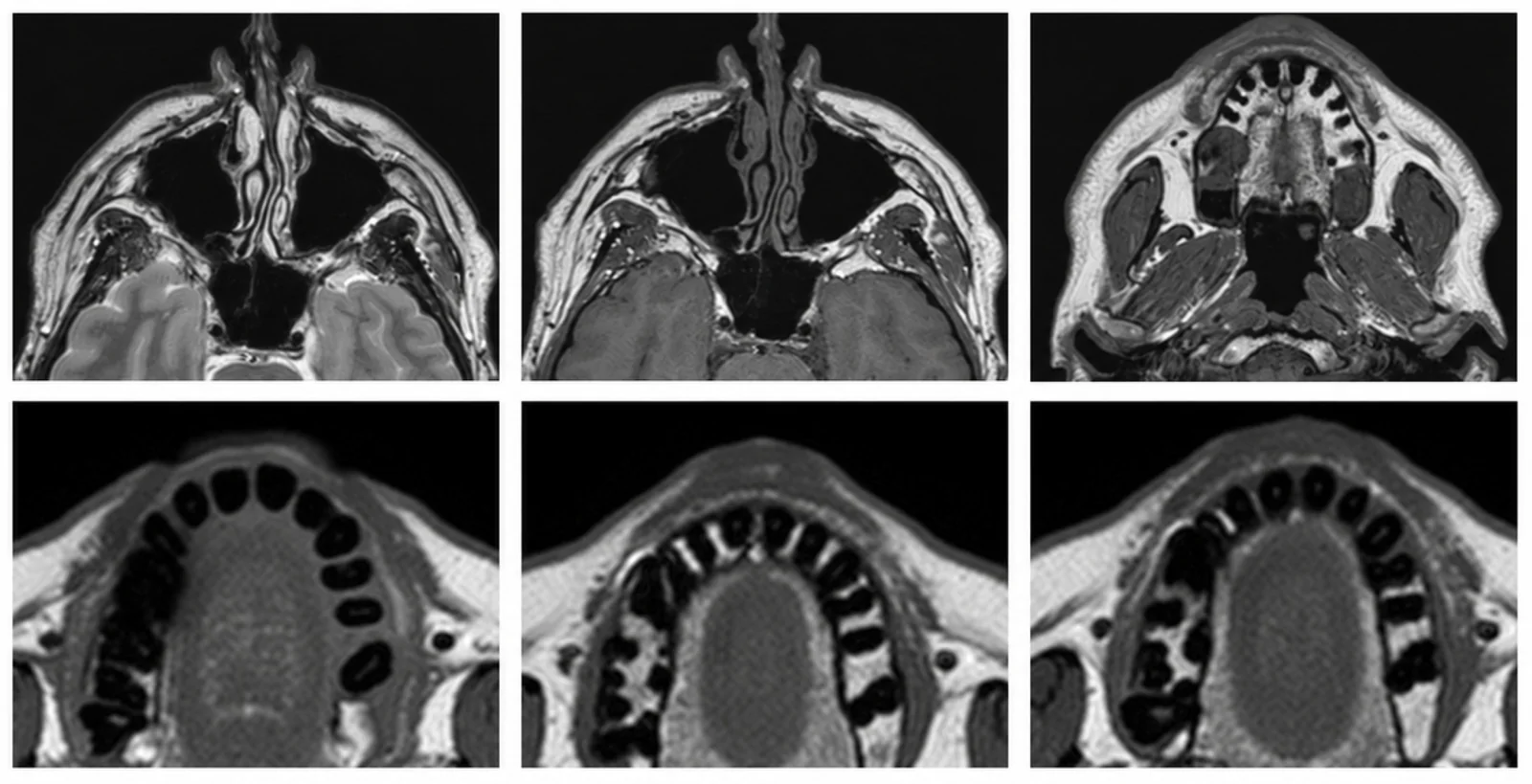
Axial magnetic resonance images of the perioral region. The upper and lower rows show representative sections through the upper and lower lips at different craniocaudal levels. The orbicularis oris forms the central muscular component of the lip and is closely related to the surrounding soft tissues; a separate SMAS-like layer is not resolved on these illustrative images. Images are original, previously unpublished, anonymized images selected from the teaching image archive of the Department of Morphology and Cell Biology, Anatomy Section, University of Oviedo, and were not obtained specifically for the present study. Complete original acquisition parameters are unavailable.

The imaging evidence should be interpreted cautiously. MRI and ultrasound demonstrate the close spatial relationship of the orbicularis oris with adjacent soft tissues, but they do not resolve the microscopic connective tissue architecture demonstrated histologically (Figure 3 and Figure 4) [21,22]. A separate SMAS-like layer was not resolved on the selected illustrative images. Given the non-standardized archival acquisition and unavailable technical parameters, this non-visualization cannot distinguish absence of a lamina from insufficient spatial resolution or unsuitable acquisition conditions and is not assigned diagnostic significance. The original imaging material presented here is solely for illustrative morphological correlation.

From a clinical perspective, this anatomical interpretation provides a rational basis for contemporary reconstructive and aesthetic procedures involving the lips. Understanding the continuity of the connective tissue framework facilitates interpretation of tissue behaviour during surgical dissection, reconstruction and minimally invasive treatments. Rather than considering the lips as a series of independent tissue layers, clinicians should recognize them as a structurally integrated fibromuscular organ whose mechanical behaviour depends upon the integrity of its connective tissue architecture.

Several limitations affect the strength and generalizability of the conclusions. Studies specifically addressing the labial SMAS are few and are generally based on small cadaveric series. Conventional histology provides predominantly two-dimensional information and may not fully represent three-dimensional relationships among muscle, connective tissue, vessels and nerves. The original histological and imaging observations included here are limited by the small number of specimens, their qualitative nature, the absence of quantitative analysis, and the potential selection bias inherent in selected archival material. The literature search was narrative rather than systematic. These limitations preclude definitive demonstration of a discrete labial SMAS and require the proposed regional-equivalent interpretation to remain a testable anatomical hypothesis.

Overall, the most consistent finding is not a classical laminar SMAS but a distinctive three-dimensional connective tissue organization extending from mucosa to skin and intimately associated with the orbicularis oris. Whether this organization is ultimately accepted as the regional equivalent of the facial SMAS will require direct three-dimensional anatomical confirmation.

## 10. Future Perspectives

The principal unresolved question is the three-dimensional architecture of the connective tissue network extending through the lip and its relationship to the orbicularis oris, mucosa and skin. Future studies should test directly whether this organization fulfils the anatomical criteria for a regional equivalent of the facial SMAS.

A major priority will be the application of three-dimensional anatomical approaches capable of reconstructing the connective tissue network while preserving its native spatial organization. Tissue-clearing methods, volumetric histology and high-resolution digital reconstruction, integrated with MRI and ultra-high-frequency ultrasound, may allow direct correlation between microscopic architecture and in vivo tissue organization.

Further studies should characterize the influence of age, sex and individual anatomical variation on the labial connective tissue framework. In particular, age-related extracellular matrix remodelling deserves investigation because of its potential relevance to changes in lip morphology and to reconstructive and aesthetic practice.

Direct correlation between connective tissue architecture and biomechanical behaviour represents another major research priority. Experimental approaches capable of relating tissue deformation to connective tissue organization, muscular activity and the mechanical environment of sensory receptors would help determine which of the functional interpretations proposed here can be demonstrated experimentally.

## 11. Conclusions

The available evidence does not establish a classical, readily separable laminar SMAS within the lips. Instead, the available anatomical evidence supports a three-dimensional connective tissue network extending from the oral mucosa to the skin and intimately incorporating the orbicularis oris muscle.

This distinctive organization may represent the regional equivalent of the SMAS in the lips rather than a classical SMAS layer. The interpretation is supported by convergent anatomical and histological observations but is not yet demonstrated conclusively by three-dimensional methods.

Biomechanical and sensory consequences of this organization remain implications rather than experimentally established functions. Further three-dimensional anatomical studies are required to define its architecture and determine its relationship to the facial SMAS with greater precision.

## Figures and Tables

**Table 1:** Principal publications informing the interpretation of a labial SMAS.

Reference	Region/Material	Methods	Direct Observations	Interpretation and Limitations
Pensler et al. [9]	Upper lip; cadaveric material	Dissection and histology	Connective tissue associated with orbicularis oris and superficial tissues	Direct anatomical evidence; limited series and no three-dimensional reconstruction
Jost et al. [10]	Upper lip; brief letter	Methodological details not sufficiently reported	Commentary supporting an upper-lip SMAS interpretation	Interpretative contribution; not an independently assessable full anatomical study
Ghassemi et al. [11]	Multiple facial regions, including the perioral region	Dissection, conventional histology, polarized-light and confocal microscopy	Regional variation in SMAS architecture; dense fibromuscular pattern periorally	Supports regional specialization; not focused exclusively on the lips
Hînganu et al. [12]	Human lips and perioral region	Gross anatomy, histology and radiological imaging	Integration of superficial fascia with orbicularis oris near the lips	Multimodal evidence; limited material and no definitive three-dimensional demonstration

## Data Availability

The original anatomical, histological, and imaging material used to provide qualitative morphological correlation in this review forms part of institutional research and teaching collections and is subject to applicable ethical and institutional regulations. Further information regarding the material may be obtained from the corresponding author upon reasonable request, subject to these regulations.

## Abbreviations

SMAS	Superficial musculoaponeurotic system
MR	Magnetic resonance
